# Representativeness of Black Patients in Cancer Clinical Trials Sponsored by the National Cancer Institute Compared With Pharmaceutical Companies

**DOI:** 10.1093/jncics/pkaa034

**Published:** 2020-04-24

**Authors:** Joseph M Unger, Dawn L Hershman, Raymond U Osarogiagbon, Anirudh Gothwal, Seerat Anand, Arvind Dasari, Michael Overman, Jonathan M Loree, Kanwal Raghav

**Affiliations:** p1 SWOG Statistics and Data Management Center, Fred Hutchinson Cancer Research Center, Seattle, WA, USA; p2 Columbia University, New York, NY, USA; p3 Multidisciplinary Thoracic Oncology Program, Baptist Cancer Center, Memphis, TN, USA; p4 Baylor University, Houston, TX, USA; p5 Department of Gastrointestinal Medical Oncology, MD Anderson Cancer Center, Houston, TX, USA; p6 BC Cancer, Vancouver, British Columbia, Canada

## Abstract

**Background:**

Many clinical trials supporting new drug applications underrepresent minority patients. Trials conducted by the National Cancer Institute’s National Clinical Trial’s Network (NCTN) have greater outreach to community sites, potentially allowing better representation. We compared the representation of Black patients in pharmaceutical company–sponsored cancer clinical trials with NCTN trials and with the US cancer population.

**Methods:**

We established a large cohort of study publications representing the results of trials that supported new US Food and Drug Administration drug approvals from 2008 to 2018. NCTN trial data were from the SWOG Cancer Research Network. US cancer population rates were estimated using Surveillance, Epidemiology, and End Results survey data. We compared the proportion of Black patients by enrollment year for each cancer type and overall. Tests of proportions were used. All statistical tests were 2-sided.

**Results:**

A total 358 trials (pharmaceutical company–sponsored trials, 85; SWOG trials, 273) comprised of 93 825 patients (pharmaceutical company–sponsored trials, 46 313; SWOG trials, 47 512) for 15 cancer types were analyzed. Overall, the proportion of Black patients was 2.9% for pharmaceutical company–sponsored trials, 9.0% for SWOG trials, and 12.1% for the US cancer population (*P* < .001 for each pairwise comparison). These findings were generally consistent across individual cancer types.

**Conclusions:**

The poor representation of Black patients in pharmaceutical company–sponsored trials supporting new drug applications could result in the use of new drugs with little data about efficacy or side effects in this key population. Moreover, because pharmaceutical company–sponsored trials test the newest available therapies, limited access to these trials represents a disparity in access to potential breakthrough therapies.

The conduct of clinical trials is vital for advancing experimental therapies to routine practice. The rapid adoption of new therapies depends on the confidence that patients, providers, and payers have in the validity of clinical trial findings, including their applicability to patients of different sociodemographic backgrounds. This foundational evidence is weakened when clinical trials are not representative of the cancer population. Yet trials supporting new drug applications to the US Food and Drug Administration (FDA), which are primarily sponsored by pharmaceutical companies, underrepresent minority patients (1, 2). In contrast, the National Cancer Institute’s (NCI’s) National Clinical Trials Network (NCTN) addresses more expansive research questions, including comparisons of different treatment combinations, treatment modalities, or combinations of multiple approved drugs in other cancers (3). These trials are designed to serve the diverse needs of cancer patients, and—like pharmaceutical company–sponsored trials—frequently inform care guidelines (4).

Given this focus, an important question is whether NCTN trials differ in representation of sociodemographic groups, and further, whether either trial process (pharmaceutical or NCTN) adequately represents minority groups compared with the US cancer population. To address this, we examined the representation of Black patients in pivotal pharmaceutical company–sponsored drug trials, NCTN trials, and the US cancer population in the same cancers over the same timeframe.

## Methods

### Data

We examined the representation of Black patients in pivotal pharmaceutical company–sponsored drug trials, NCTN trials, and the US cancer population in the same cancers over the same timeframe using a cohort design (5). We reviewed all FDA drug approvals based on pharmaceutical company–sponsored trials for hematologic and solid tumor cancers from 2008 to 2018, as previously described (6). Trials supporting these approvals were identified using PubMed and ClinicalTrials.gov (1). Corresponding primary study publications must have reported enrollment by race, including Black patients. Data representing NCTN trials were obtained from the SWOG Cancer Research Network, which conducts treatment trials in all major cancers. We included initial enrollments to treatment trials, including all patients from trials led by SWOG, or patients enrolled by SWOG investigators to trials led by other NCTN groups in which SWOG participated. US cancer population rates were estimated using data from the Surveillance, Epidemiology, and End Results registry (7).

This study followed the Strengthening the Reporting of Observational Studies in Epidemiology reporting guideline (5).

Institutional review board approval for inclusion of data from pharmaceutical company–sponsored trials was not required because these data were from publicly available published reports that included no identifiable patient-level data. Each SWOG trial included in this secondary analysis was previously approved by an institutional review board, and written informed consent was previously obtained from all patients.

### Statistical Methods

We combined pharmaceutical company–sponsored trials by cancer type. Enrollment years for these trials were not routinely available from study publications. Enrollment duration was assumed to be 3 years, and trial maturation, analysis, and reporting were assumed to require 1 year; thus, the assumed enrollment years to these trials were 2004 (the first assumed year of enrollment for a trial published in 2008) to 2017 (the last assumed year of enrollment for a trial published in 2018).

For pharmaceutical company–sponsored trials, for each cancer type, we calculated the proportion of Black patients by enrollment year. Corresponding estimates for SWOG trials were derived using a 3-year average window (±1 year) about the year of pharmaceutical company–sponsored trial enrollment due to sparse samples for some cells. Given the ±1-year window, the total enrollment period for SWOG trials was 2003–2018. For cells with fewer than 10 SWOG patients, mean imputation was used for the proportion of Black patients within the enrollment year. We adjusted Surveillance, Epidemiology, and End Results registry rates to reflect the US cancer population by weighting estimates based on national distributions of age, sex, and race using US Census data, as previously described (7, 8). Summary overall and cancer-specific estimates for all groups (pharmaceutical company–sponsored trials, SWOG trials, and US cancer population) were weighted by enrollment in the pharmaceutical company–sponsored trials by year to enable a consistent comparison across groups.

Comparisons between groups were conducted using binomial tests of proportions, with the US cancer population estimates considered fixed. All *P* values ≤.05 were considered statistically significant. All statistical tests were 2-sided.

## Results

### Study Inclusion

**Figure 1.**
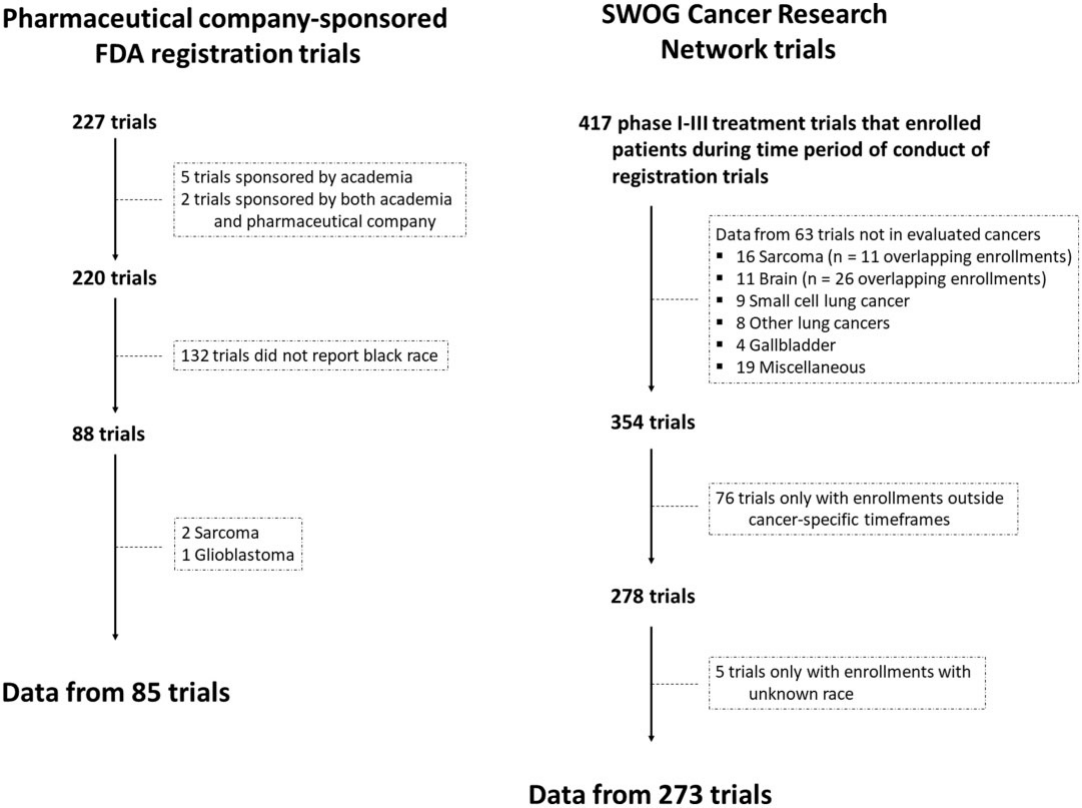
Trial inclusion diagram.

Among the 227 FDA registration trials for malignancy, 220 (96.9%) were pharmaceutical company sponsored (Figure 1). The majority (132, 60.0%) did not report Black race and were excluded. There were 417 SWOG phase I-III treatment trials that enrolled patients during the corresponding time period. Among these, enrollment data from 63 trials were excluded. Although 2 sarcoma trials and 1 glioblastoma trial were included in the pharmaceutical company–sponsored FDA registration trial database, only 11 enrollments from SWOG sarcoma trials and 26 enrollments from SWOG brain trials were available in the overlapping time periods; given small numbers, no comparisons in Black enrollment between pharmaceutical company–sponsored trials and SWOG trials were conducted for these 2 cancers. For the remaining SWOG cancers, no corresponding pharmaceutical company–sponsored registration trial was available for comparison. Data from 76 other SWOG trials with no enrollments within the cancer-specific enrollment timeframe for the pharmaceutical company–sponsored FDA registration trials were excluded.

### Study and Patient Characteristics

Overall, we examined data from 85 pharmaceutical company–sponsored trials with 46 313 patients and 273 SWOG trials with 47 512 patients (93 825 total patients) in 15 separate cancers (Table 1). Common cancers (breast, colorectal, lung, prostate) were 42.4% of studies and 59.9% of enrollments for pharmaceutical company–sponsored FDA registration trials and 46.5% of studies and 72.4% of enrollments for SWOG trials. SWOG CRN = SWOG Cancer Research Network

**Table 1.** Characteristics of the pharmaceutical company-sponsored pivotal trials and SWOG Cancer Research Network trials*

Cancer type	Trials, No. (%)		Sample size, No. (%)	
	Pharma	SWOG CRN	Pharma	SWOG
Bladder	3 (3.5)	5 (1.8)	710 (1.5)	946 (2.0)
Breast	9 (10.6)	51 (18.7)	8178 (17.7)	23 098 (48.9)
Colorectal	4 (4.7)	11 (4.0)	2706 (5.8)	2417 (5.1)
Gastroesophageal	3 (3.5)	10 (3.7)	1208 (2.6)	632 (1.3)
Gynecologic	2 (2.4)	5 (1.8)	371 (0.8)	144 (0.3)
Head and neck	3 (3.5)	12 (4.4)	794 (1.7)	98 (0.2)
Leukemia	8 (9.4)	34 (12.5)	3066 (7.0)	3632 (7.6)
Liver	2 (2.4)	2 (0.7)	787 (1.7)	101 (0.2)
Lung	18 (21.2)	41 (15.0)	11 322 (26.0)	3902 (8.2)
Lymphoma	9 (10.6)	31 (11.4)	2408 (5.2)	2493 (5.2)
Melanoma	4 (4.7)	11 (4.0)	906 (2.1)	978 (2.1)
Myeloma	7 (8.2)	14 (5.1)	3449 (7.9)	1340 (2.8)
Pancreas	2 (2.4)	9 (3.3)	1398 (3.0)	335 (0.7)
Prostate	5 (5.9)	24 (8.8)	5551 (12.7)	4985 (10.5)
Renal	6 (7.1)	13 (4.8)	3409 (7.8)	2411 (5.1)
Total No.	85	273	46 313	47 512

*SW OG CRN = SWOG Cancer Research Network

Pharmaceutical company–sponsored trials were more likely to be phase III studies (63.5% vs 49.1%), although in both research settings most patients were from phase III trials (pharmaceutical company–sponsored trials, 86.5%; SWOG trials, 83.6%). SWOG-led studies comprised 48.4% of all included NCTN trials (132 of 273) and 78.5% of all enrolled patients (37 293 of 47 512).

### Overall Estimates

**Figure 2.**
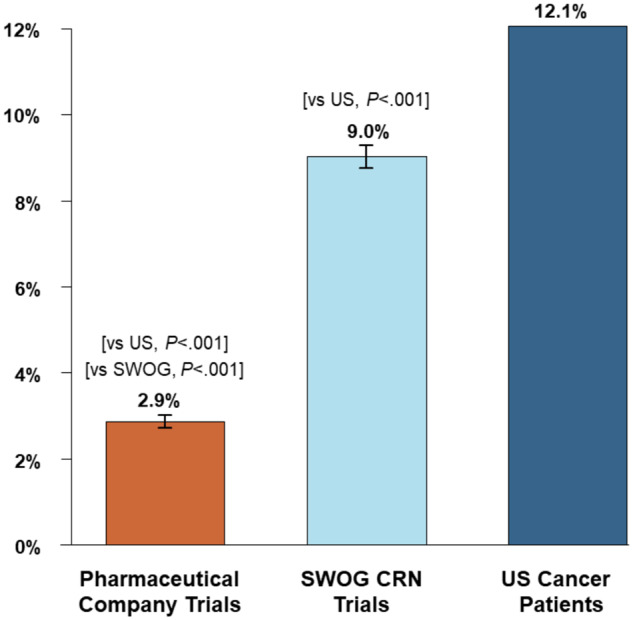
Proportion of Black patients by research setting and for the US cancer population. *P* values are from a test of proportions. No error bars are indicated for US cancer patients because these rates are considered fixed population rates rather than sample estimates. SWOG CRN = SWOG Cancer Research Network.

Overall, the proportion of Black patients was 2.9% for pharmaceutical company–sponsored trials, 9.0% for SWOG trials, and 12.1% for the US cancer population (*P* < .001) for each pairwise comparison (Figure 2).

### Cancer-Specific Estimates in Common Cancers

**Figure 3.**
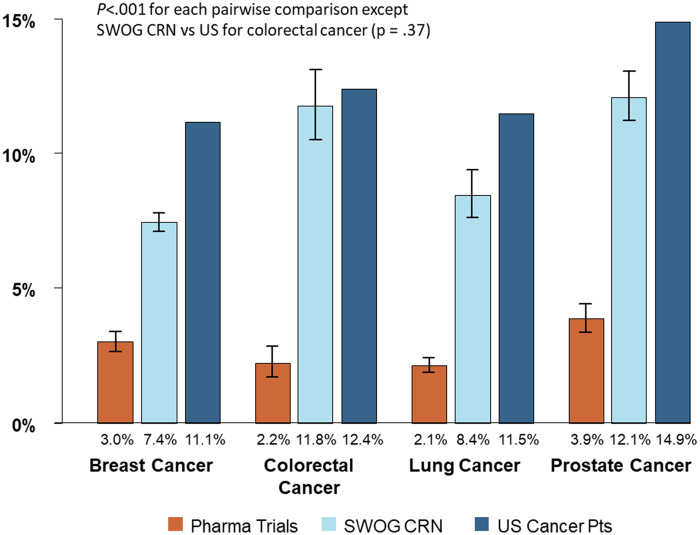
Proportion of Black patients by research setting and for the US cancer population for common cancers. The vertical lines indicate the 95% confidence intervals for the pharmaceutical company–sponsored trial estimates and for the SWOG Cancer Research Network trial estimates, respectively. No error bars are indicated for US cancer patients because these rates are considered fixed population rates rather than sample estimates.

Among common cancers (breast, colorectal, lung, and prostate), the proportion of Black patients was much lower for pharmaceutical company–sponsored trials than for either SWOG trials or for the US cancer population (*P* < .001 for each pairwise comparison; Figure 3). For instance, for breast cancer, the proportion of Black patients was 3.0% for pharmaceutical company–sponsored trials compared with 7.4% for SWOG trials and 11.1% for the US cancer population. Similarly, for prostate cancer, the proportion of Black patients was 3.9% for pharmaceutical company–sponsored trials compared with 12.1% for SWOG trials and 14.9% for the US cancer population.

**Figure 4.**
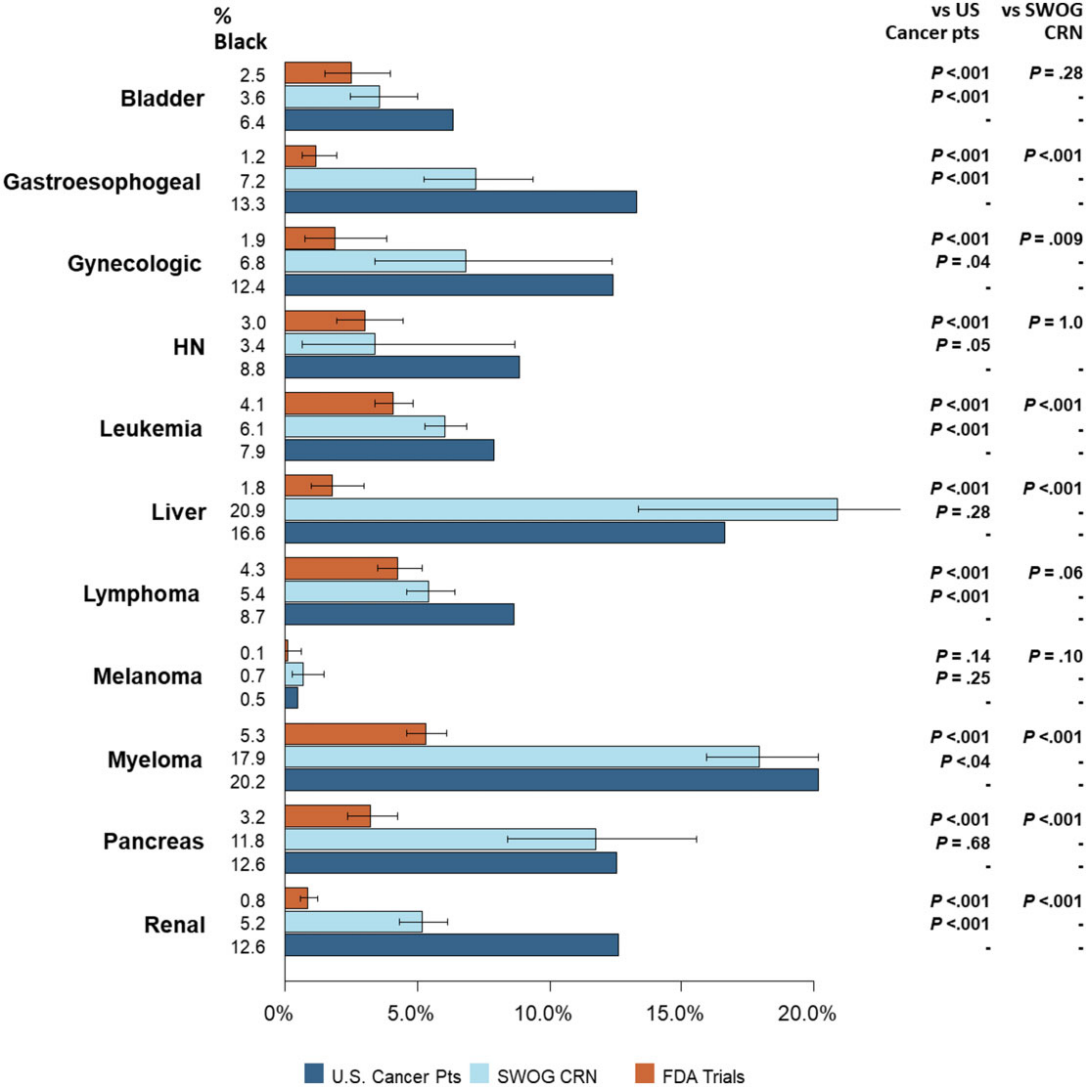
Proportion of Black patients by research setting and for the US cancer population for other cancers. The horizontal lines indicate the 95% confidence intervals for the pharmaceutical company–sponsored trial estimates and for the SWOG Cancer Research Network trial estimates, respectively. No error bars are indicated for US cancer patients because these rates are considered fixed population rates rather than sample estimates.

The proportion of Black patients was also lower for SWOG trials compared with the US cancer population for each of the most common cancers except colorectal cancer (11.8% vs 12.4%, *P* = .37; Figure 3). SWOG CRN = SWOG Cancer Research Network.

### Cancer-Specific Estimates in Other Cancers

For each of the other cancer types, the proportion of Black patients was statistically significantly lower for pharmaceutical company–sponsored trials compared with the US cancer population (*P* < .001 in each instance), except for melanoma (0.1% vs 0.5%, *P* = .14; Figure 3). For instance, the proportion of Black patients in pharmaceutical company–sponsored trials was much lower compared with the US cancer population for liver cancer (1.8% vs 16.6%, *P* < .001) and for myeloma (5.3% vs 20.2%, *P* < .001).

Similarly, the proportion of Black patients was statistically significantly lower (*P* < .05) for pharmaceutical company–sponsored trials compared with SWOG trials for all cancers except bladder (2.5% vs 3.6%, *P* = .28), head and neck (3.0% vs 3.4%, *P* = 1.0), lymphoma (4.3% vs 5.4%, *P* = .06), and melanoma (0.1% vs 0.7%, *P* = .10).

The proportion of Black patients was statistically significantly lower (*P* < .05) for SWOG trials compared with the US cancer population for all cancers with 3 exceptions. The proportion of Black patients was not different between groups for liver cancer (20.9% vs 16.6%, *P* = .28), melanoma (0.7% vs 0.5%, *P* = .25), and pancreatic cancers (11.8% vs 12.6%, *P* = .74). Furthermore, although the proportion of Black patients in SWOG trials of myeloma cancer was statistically significantly lower than the rate in the US cancer population, the absolute difference in proportions between the groups was not large (17.9% vs 20.2%, *P* = .04).

## Discussion

This analysis reaffirms prior evidence that Black patients are severely underrepresented in pharmaceutical company–sponsored pivotal trials compared with the US cancer population; fewer than 1 in 4 of the expected number of Black patients were enrolled (2.9% vs 12.1%) (1, 2). Black representation in pharmaceutical company–sponsored trials was also far below that of NCTN trials. These patterns were consistent across 15 cancer types. These findings demonstrate that pharmaceutical company–sponsored trials for new drug applications include few Black patients and suggest that the existing trial program of the NCTN offers an alternative model to improve minority recruitment.

The FDA, in partnership with the American Association for Cancer Research, is currently examining ways to improve representation of Black patients in FDA registration trials. Although their focus is on trials for myeloma—given the high prevalence of myeloma in the Black population (9)—models for improving minority participation could be extended to other cancer settings. One potential model already exists in the form of federally sponsored trials conducted by the NCI’s network groups in which Black enrollment was 3 times higher compared with pharmaceutical company–sponsored trials (9.0% vs 2.9%), including for myeloma (17.9% vs 5.3%, *P* < .001). Several possibilities may explain this. Consistent with the mission to serve the broader community of cancer patients, a particular emphasis of the NCTN trial program is the participation of patients from both large academic centers and community-based sites (3, 10). As noted in a previous Institute of Medicine report that focused on NCTN trial conduct in the 21st century, NCI-sponsored trials “extend participation beyond research-oriented facilities—such as academic medical centers and cancer centers—to community hospitals” in order to evaluate therapies “in settings more representative of current medical practice” and to better ensure “patient access to innovative therapies in settings other than academic medical centers or cancer centers.” (3) In contrast, pharmaceutical company–sponsored trials mostly target large academic centers. These centers are likely harder to access for socioeconomically disadvantaged groups due to requirements to travel or take time off work (11, 12). During the time period represented in this analysis, SWOG has been (and continues to be) a member of the NCI’s Community Oncology Research Program and its antecedent, the Community Clinical Oncology Program. These programs are designed to provide access to clinical trials for community sites and patients and include minority or underserved sites that serve populations with 30% or more racial or ethnic minorities or rural residents; these programs have contributed about one-third of patients to NCTN trials (13). Pharmaceutical companies could improve racial or ethnic diversity in their trials—and expand access to all patients—by emulating outreach to community and minority or underserved community sites.

The NCTN model alone may not be sufficient; we found that NCTN trials also underrepresented Black patients (9.0% vs 12.1%). This observation stands in contrast to studies showing representative enrollment of Black patients in SWOG treatment trials in past periods (through at least 2007) (8, 14, 15). An extensive literature has documented the numerous social and economic barriers potential research participants face to participation in trials, both overall and for Black patients in particular, including structural, clinical, financial, communication, literacy, and trust barriers (16–18). Many of these problems endure despite the efforts of providers and researchers to extend opportunities for trial participation to disadvantaged groups through such mechanisms as increased community-driven research, enlistment of community leaders, peer recruitment, targeted media and marketing, and a focus on cultural competency (19–27).

The more recently observed underrepresentation in the proportion of Black patients in SWOG trials compared with the US cancer population may be due to changes in the conduct of cancer treatment trials, such as the increased use of biomarker-specific eligibility criteria for targeted therapy trials, which could create additional barriers to access for disadvantaged populations (28, 29). Future work is warranted to confirm whether Black enrollment to NCTN trials has decreased over time and to identify potential causes for any decrease. A trend towards worse Black enrollment in NCTN trials would be especially troubling given recent efforts to expand clinical trial access through reductions in unnecessary trial exclusion criteria (30). In sum, there remains a meaningful opportunity to improve diverse enrollment to NCTN trials.

One other reason that few patients in pharmaceutical company–sponsored trials in oncology are Black is that these trials frequently recruit patients from international sites. The FDA’s annual Drug Trials Snapshot Report for 2018 showed that among the n = 5157 patients enrolled in trials supporting 17 new drugs in oncology, only 36% were from US sites (31). This raises the difficult question of whether—if racial and ethnic representativeness is indeed a vital concern for FDA registration trials—trial enrollment should focus more commonly on domestic patient enrollment.

This study was limited by the fact that few pharmaceutical company–sponsored trials report enrollment for other racial or ethnic groups, so we were unable to extend the analysis to include other demographic groups, including Hispanics. Fuller reporting of racial or ethnic composition in pharmaceutical company–sponsored trials is needed. This is especially important because pharmaceutical company–sponsored trials often recruit from international sites, which may actually make those trials more representative than NCTN trials for certain patient groups (eg, Asians) (1). Finally, this study focused mainly on enrollment in SWOG trials, so the results may not be fully generalizable to other NCTN groups.

The poor representation of Black patients in pharmaceutical company–sponsored pivotal trials is of vital scientific interest. The concern is that these trials could support use of new drugs with little data about efficacy or side effects in this key population. Because pharmaceutical company–sponsored trials test the newest available therapies—which are increasingly driven by well-characterized mechanisms of action, increasing their likelihood of success while reducing drug-related toxicity—limited access to these clinical trials represents a disparity in access to potential breakthrough therapies.

### Funding

Research reported in this publication was supported in part by the Division of Cancer Prevention, National Cancer Institute, National Institutes of Health under grant award 5UG1CA189974 (DLH) and 2UG1CA189873-06 (RUO); in part by the Hope Foundation for Cancer Research in support of infrastructure data analysis and trial design within SWOG, at the SWOG Statistical and Data Management Center (JMU); and in part by the Michael Smith Health Professional Investigator program (JML).

### Notes

**Role of the funders:** The funders had no role in the design of the study; the collection, analysis, and interpretation of the data; the writing of the manuscript; and the decision to submit the manuscript for publication.

**Conflicts of interest:** Drs Unger, Hershman, Gothwal, Anand, Dasari, Overman, and Raghav report no conflicts. Dr Osarogiagbon reports Astra Zeneca, Eli Lilly, Foundation Medicine, Pfizer, Association of Community Cancer Centers, American Cancer Society, patents for a lymph node specimen collection kit and founding interest in Oncobox Device, Inc. Dr Loree reports IPSEN Canada, Amgen, Bayer, Taiho, Novartis (personal fees).
